# A broader active site in *Pyrococcus horikoshii* CoA disulfide reductase accommodates larger substrates and reveals evidence of subunit asymmetry

**DOI:** 10.1002/2211-5463.12439

**Published:** 2018-06-09

**Authors:** Kevin Sea, Jerry Lee, Daniel To, Berniece Chen, Matthew H. Sazinsky, Edward J. Crane

**Affiliations:** ^1^ Department of Chemistry Pomona College Claremont CA USA; ^2^ Department of Wine Studies Santa Rosa Junior College CA USA; ^3^ Department of Biology Pomona College Claremont CA USA

**Keywords:** CoA disulfide, coenzyme A‐disulfide reductase, elemental sulfur metabolism, half‐site reactivity, polysulfide

## Abstract

Within the family of pyridine nucleotide disulfide oxidoreductase (PNDOR), enzymes are a group of single‐cysteine containing FAD‐dependent reductases that utilize a tightly bound coenzyme A to assist in the NAD(P)H‐dependent reduction of di‐, per‐, and polysulfide substrates in bacteria and archaea. For many of these homodimeric enzymes, it has proved difficult to determine the substrate specificity and metabolic function based on sequence and genome analysis alone. Coenzyme A‐disulfide reductase (CoADR) isolated from *Pyrococcus horikoshii* (*ph*CoADR) reduces Co‐A per‐ and polysulfides, but, unlike other highly homologous members of this group, is a poor CoA disulfide reductase. The *ph*CoADR structure has a narrower access channel for CoA substrates, which suggested that this restriction might be responsible for the enzyme's poor activity toward the bulky CoA disulfide substrate. To test this hypothesis, the substrate channel was widened by making four mutations along the channel wall (Y65A, Y66A, P67G, and H367G). The structure of the quadruple mutant shows a widened substrate channel, which is supported by a fourfold increase in *k*_cat_ for the NAD(P)H‐dependent reduction of CoA disulfide and enhanced activity toward the substrate at lower temperatures. Anaerobic titrations of the enzyme with NADH revealed a half‐site reactivity not observed with the wild‐type enzyme in which one subunit of the enzyme could be fully reduced to an EH_4_ state, while the other remained in an EH_2_ or EH_2_·NADH state. These results suggest that for these closely related enzymes, substrate channel morphology is an important determinant of substrate specificity, and homology modeling will be the preferred technique for predicting function among PNDORs.

**Abbreviations**
CoADR: coenzyme A‐disulfide reductase
EH_2_: 2 electron‐reduced enzyme
EH_4_: four electron‐reduced enzyme
eq: equivalents
Nox: NAD(P)H oxidase
Nsr: NADPH: elemental sulfur oxidoreductase
phCoADR: *Pyrococcus horikoshii* coenzyme A‐disulfide reductase
PNDOR: pyridine nucleotide disulfide oxidoreductase
Psr: polysulfide reductase
saCoADR: *Staphylococcus aureus* coenzyme A‐disulfide reductase

*Pyrococcus horikoshii* coenzyme A‐disulfide reductase (*ph*CoADR; http://www.chem.qmul.ac.uk/iubmb/enzyme/EC1/8/1/14.html) is an FAD‐ and NAD(P)H‐dependent homodimeric member of the pyridine nucleotide disulfide oxidoreductase (PNDOR) family of enzymes. PNDORs, which are found in both bacteria and archaea, typically catalyze the reduction of S–S bonds in di‐, per‐, and polysulfides as well as O–O bonds in dioxygen and peroxides. The metabolic purpose of the reduction varies by enzyme and in many cases is unknown, but functions can include maintenance of cellular redox balance 1, sulfur‐based respiration 2, 3, and oxidative defense 4. Examining homologous sequences among PNDORs to identify the metabolic function and/or substrates of these enzymes generally has had poor predictive ability 4, and for an uncharacterized microbe, this often makes assigning a phenotype and assessing the availability of a metabolic pathway from the genomic information difficult. In the specific case of PNDOR enzymes, these assignments are important for understanding their role in microbial elemental sulfur cycling at the organismal and community levels.

The comparison of the PNDOR structures, however, has revealed an apparent pattern in which substrate specificity is based on the relative openness of the active‐site channel. In both *Bacillus anthracis* 4, 5 and *Staphylococcus aureus* 4, 6, the active‐site channel that admits the oxidizing substrate (i.e., sulfur, polysulfide or CoA derivatives) is relatively wide and able to efficiently accommodate the large CoA disulfide substrate. In contrast, *ph*CoADR has a relatively narrow active‐site channel, prefers polysulfide, CoA‐polysulfide, and CoA persulfide substrates, and shows no activity toward CoA disulfide (CoA‐S‐S‐CoA) at 50 °C and only moderate activity toward CoA disulfide at 75 °C 4.

In all three CoADR enzymes and the related NADH‐dependent polysulfide reductase (Npsr) from *Shewanella oneidensis* MR1 7, residues 56‐76 (*ph*CoADR numbering) form one side of the CoA‐binding cleft. We speculated previously that the CoA disulfide was excluded from the *ph*CoADR active site by the narrowing of the cleft due to the bulky side chains of Y65, Y66, and R75 and by a shift in the Cα backbone of residues 56–76 toward the opposite side of the CoA‐binding channel 4. In Archaea, where glutathione is either absent or present at very low concentrations 1, CoA disulfide has been hypothesized to replace glutathione as the small molecule regulator of intracellular redox conditions. However, the apparent exclusion of CoA disulfide and glutathione disulfide [oxidized glutathione (GSSG)] from the *ph*CoADR substrate channel suggests that this may not be the case for *P. horikoshii*, calling into question the metabolic function of this sulfur‐reducing enzyme and how intracellular redox status is maintained.

To explore the factors that control the substrate specificity of *ph*CoADR and PNDOR enzymes in general, we widened the active‐site channel of *ph*CoADR by making a Y65A, Y66A, P67G, and H367G quadruple mutant and determined whether these changes overcome the poor CoA disulfide reductase activity of the enzyme. Kinetic analysis revealed that this widening of the substrate channel resulted in improved utilization of CoA disulfide at temperatures as low as 50 °C, although the crystal structure of the enzyme revealed the backbone of the quadruple mutant outside the active site did not change significantly.

Unexpectedly, these mutations also revealed subunit asymmetry in *ph*CoADR. For wild‐type *ph*CoADR, anaerobic titrations of the oxidized enzyme with NAD(P)H show formation of a FAD‐NAD(P)H complex on each subunit, with no reduction of the enzyme‐bound FAD to FADH_2_. Such behavior is characteristic of FAD‐dependent enzymes that are not active in the reduction of O_2._ Thus, for *ph*CoADR, the reducing equivalents remain on NAD(P)H and poised to reduce substrates other than O_2._ For the quadruple mutant, the addition of excess NADH reduces FAD to FADH_2_ in only one of the two subunits, leaving the other FAD oxidized, most likely with a bound NADH. The implications of these results are discussed.

## Methods

### Cloning, overexpression, and purification of CoADR

Wild‐type *P. horikoshii* CoADR (*ph*CoADR) was expressed in BL21(DE3) *Escherichia coli* cells containing a pET24d plasmid with the gene and purified as previously described 4. A codon‐optimized gene for the quadruple mutant Y65A, Y66A, P67G, H367G (‘quad mutant’) was purchased from GenScript (Piscataway, NJ, USA) and inserted into a pET21b+ vector at the *Nde*I and *Xho*I restriction sites to create a final construct with a C‐terminal His tag. A C48S mutation was made in the quad mutant gene by site‐directed mutagenesis (Quick Change) using the forward primer 5′‐gtgtctcatgcaccgagcggcattccgtatg‐3′ and reverse primer 5′‐catacggaatgccgctcggtgcatgagacac‐3′. All constructs were sequenced to confirm accuracy. The quad mutant and the C48S‐quad mutant protein were expressed and purified according to the same procedures used for the wild‐type enzyme except the heat step was done at 60 °C. All *ph*CoADR protein concentrations were determined by measuring the amount of bound FAD (ε_460 nm_ = 10 200 m ^−1^·cm^−1^) 8.

### Steady‐state kinetic assays measuring reductase activities

Kinetic assays for all substrates were conducted in quartz cuvettes, with a total solution volume of 500 μL, by monitoring the oxidation of NAD(P)H at 340 nm (ε = 6220 m ^−1^·cm^−1^), or in the case of 5,5′‐dithiobis‐2‐nitrobenzoic acid, the appearance of the TNB product at 412 nm (ε = 13 600 m ^−1^·cm^−1^). The temperature trial assays were conducted with 100−400 nm purified *ph*CoADR in 50 mm potassium phosphate buffer, pH 7.5 (as measured during pH adjustment at room temperature). NAD(P)H and substrates were preincubated in the assay buffer until they came to the assay temperature, followed by addition of enzyme. Coenzyme A‐disulfide was purchased from Sigma‐Aldrich (St. Louis, MO, USA). The slow NADH and NADPH‐dependent background oxidase activities observed were subtracted from each assay. All reactions showed a direct dependence of observed rate on enzyme concentration.

### Anaerobic titrations

Anaerobic titrations were conducted as described previously 9. Absorbance characteristic of FAD‐ and NADH‐dependent disulfide reductase enzymes include NADH (λ_max_ at 340 nm; ε = 6220 m ^−1^·cm^−1^), FAD (λ_max_ at approximately 450 nm; ε = 10 220 m ^−1^·cm^−1^), a charge transfer between the active‐site Cys‐S‐ and FAD (λ_max_ at 500–550 nm) and a charge transfer between FADH_2_ and NADP^+^ (λ_max_ at 750 nm) 10. Titrations were performed in duplicate, with representative titrations presented.

### Structure determination

Purified *ph*CoADR quad mutant was exchanged into 50 mm MOPS, pH 7.0, and 5% glycerol buffer and concentrated. The protein was crystallized using the sitting drop vapor diffusion method at room temperature by combining equal volumes of protein at 5–10 mg·mL^−1^ and mother liquor comprising 75–100 mm sodium acetate, pH 4.7, 50–100 mm ammonium acetate, 4–8% PEG 8000, and 0.01% sodium azide. Before data collection, the crystals were harvested and flash‐frozen with liquid nitrogen in a cryo‐solution comprising the mother liquor and 25% glycerol.

Low‐resolution diffraction data were collected at SSRL on BL12‐2 to 3.6 Å. The crystals belonged to the space group *R*3 with unit cell dimensions of *a* = *b* = 191.96 Å and *c* = 74.87 Å. A single homodimer comprised the asymmetric unit. The data were indexed and scaled using XDS 11 and Scala 12, respectively. Molecular replacement was performed using Molrep and the wild‐type CoADR structure (4F9X). The model was refined using Refmac5 13 in the CCP4 suite of programs and manually built in Coot 14. Given the low data to parameter ratio, the structure was refined by using B‐group factors, reference model restraints to 4F9X, secondary structure restraints, NCS and TLS parameters. All data processing and model refinement statistics are shown in Table 1. Structural validation in COOT indicated 95.8% and 3.8% of residues fell into preferred and allowed regions of a Ramachandran plot, respectively.

**Table 1:** X‐ray data collection, phase determination, and refinement statistics for the *ph*CoADR Quad Mutant

Data collection	
Beamline	SSRL 12‐2
Wavelength (Å)	0.98
Space group	*R*3
Unit cell dimensions (Å)	191.96 × 191.96 × 74.87
Resolution range (Å)	39.3–3.6 (3.73–3.60)
Total reflections	62 091
Unique reflections	11 879 (1190)
Completeness (%)^a^	99.4 (96.8)
Multiplicity	5.2 (4.8)
*I*/σ (*I*)^a^	22.8 (3.0)
*R*_sym_ (%)^a^^,^ b	4.1 (46.6)
Refinement	
*R*_cryst_ (%)^c^	25.8
*R*_free_ (%)^d^	29.1
r.m.s deviation bond length (Å)	0.010
r.m.s deviation bond angles (°)	1.756
No. of protein atoms	6821
No. of nonprotein atoms	202
Water molecules	0
RMSD (Å^2^) to wt CoADR (4F9X)	0.178
PDB Code	http://www.rcsb.org/pdb/search/structidSearch.do?structureId=5L1N

^a^ Values n parentheses are for the highest resolution shell. ^b^ *R*_sym_ = Σ_*i*_Σ_*hkl*_|*I*_*i*_(*hkl*) − <*I*(*hkl*)>|/Σ_*hkl*_<*I*(*hkl*)>, where *I*_*i*_(*hkl*) is the *i*th measured diffraction intensity and <*I*(*hkl*)> is the mean intensity for the Miller index (*hkl*). ^c^ *R*_cryst_ = Σ_*hkl*_||*F*_o_(*hkl*)| − |*F*_c_(*hkl*)||/Σ_*hkl*_|*F*_o_(*hkl*)|. ^d^ *R*_free_ = *R*_cryst_ for a test set of reflections (5% in each case).

## Results

### Low‐resolution structure of the quadruple mutant

**Figure 1:**
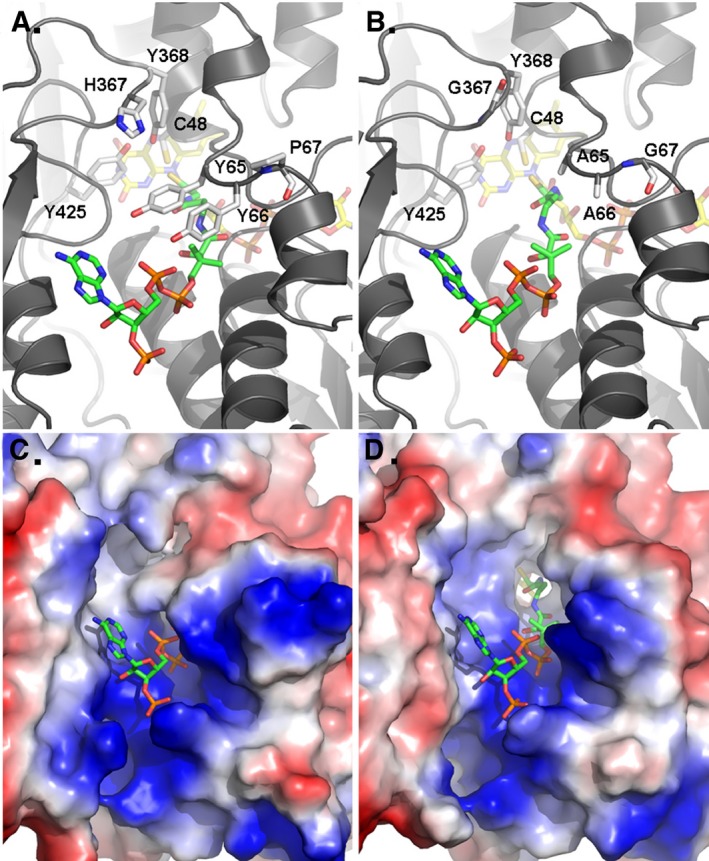
Crystal Structure of phCoADR wild‐type (http://www.rcsb.org/pdb/search/structidSearch.do?structureId=4FX9) and Quad Mutant (http://www.rcsb.org/pdb/search/structidSearch.do?structureId=5L1N). (A) The wild‐type *ph*CoADR CoA‐binding site. (B) Quad mutant *ph*CoADR CoA‐binding pocket. (C) Electrostatic surface of the CoA‐binding site. (D) Electrostatic surface representation of the Quad mutant CoA‐binding site. In (C) and (D), CoA is depicted as sticks where carbon is green, nitrogen blue, oxygen red, and phosphate orange.

**Figure 2:**
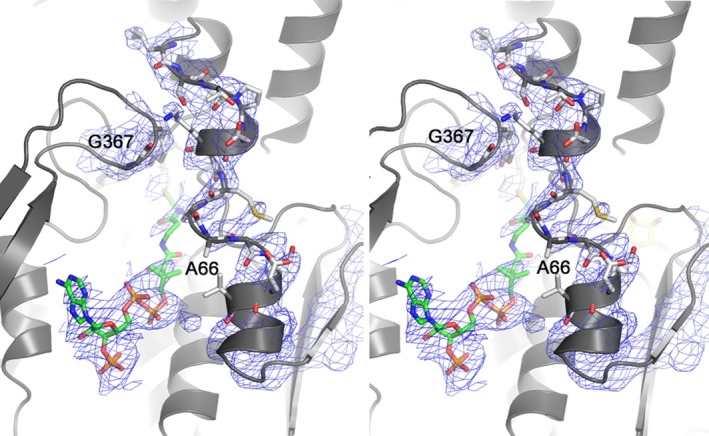
Stereoview depicting 2|*F*_o_|−|*F*_c_| electron density maps contoured to 1 around residues 54–80 and 366–368 in the *ph*CoADR Quad mutant.

A crystal structure of the quadruple mutant was obtained at a resolution of 3.6 Å. While resolution is not high enough to make conclusions based on the specific positioning of atoms, it does provide valuable information with regard to the gross structure. The backbone positioning of the wild‐type and quadruple mutant enzymes were similar and had an r.m.s.d between Cα atoms of 0.28 Å^2^ (Fig. 1A, B). As noted before, residues 56–76 comprise a significant portion of the channel and among the different homologs the backbone of this segment varies in distance from the opposite side of the channel. H367 is located on the channel surface, just opposite to Y65 and directly above the catalytic cysteine (C48). Y65, Y66, P67, and H367 are not conserved among PNDOR family members or among the subclass of putative sulfur‐metabolizing hyperthermophiles. As expected, converting the bulky side chains of Y65, Y66, and H367 to alanine confers more empty volume in the CoA‐binding channel (Fig. 1). The electron density of the protein backbone in the area around the P67G mutation is relatively weak and suggests greater disorder in this region compared to what was previously observed in the crystal structure of the wild‐type enzyme (Fig. 2). This observation is consistent with a proline to glycine mutation allowing for a greater number of favorable ψ and φ dihedral angles and therefore more flexibility in this region of the CoA‐binding channel.

### Steady‐state kinetics of wild‐type and quad mutant phCoADR

Steady‐state kinetics of CoA disulfide reduction was examined to determine whether the mutations increased space in the active‐site pocket to allow access of the CoA disulfide substrate (Table 2). When either NADH or NADPH is used in steady‐state assays, the *k*_cat_ for the quadruple mutant is ~ 4‐fold higher than the wild‐type enzyme (Table 2). For both enzymes, *K*_m_ for NADPH is much lower than the *K*_m_ for NADH, reflecting a clear substrate preference for NADPH. Despite the mutations that widened the CoA‐binding pocket, at 75 °C the *K*_m_ for CoA disulfide is nearly identical in both the wild‐type and mutant enzymes. To further test the plasticity of the substrate channel, assays were run using glutathione disulfide as the substrate. Neither enzyme exhibited activity with GSSG 4.

**Table 2:** Steady‐state kinetics of wild‐type and mutant *ph*CoADR at 75 °C, 50 mm potassium phosphate, pH 7.5

Enzyme	Constant substrate (100 μm)	Varied substrate	*k*_cat_ (s^−1^)	*K*_m_ (μm)
Wild‐type	CoA disulfide	NADPH	5.8 ± 0.2	< 9 (limit) (NADPH)
Quad mutant	CoA disulfide	NADPH	21.6 ± 2.3	16.4 ± 4.9 (NADPH)
Wild‐type	NADPH	CoA disulfide	4.4 ± 1.3	52 ± 1 (CoA disulfide)
Quad mutant	NADPH	CoA disulfide	18.8 ± 2.8	57.0 ± 6.4 (CoA disulfide)
Wild‐type	CoA disulfide	NADH	2.3 ± 0.3	73 ± 12 (NADH)
Quad mutant	CoA disulfide	NADH	8.1 ± 1.4	75 ± 6 (NADH)
Wild‐type	NADH	CoA disulfide	5.43 ± 0.48	28.6 ± 4.5 (CoA disulfide)
Quad mutant	NADH	CoA disulfide	1.80 ± 0.17	86.0 ± 5.9 (CoA disulfide)

### Temperature dependence of wild‐type and quadruple mutant phCoADR

**Figure 3:**
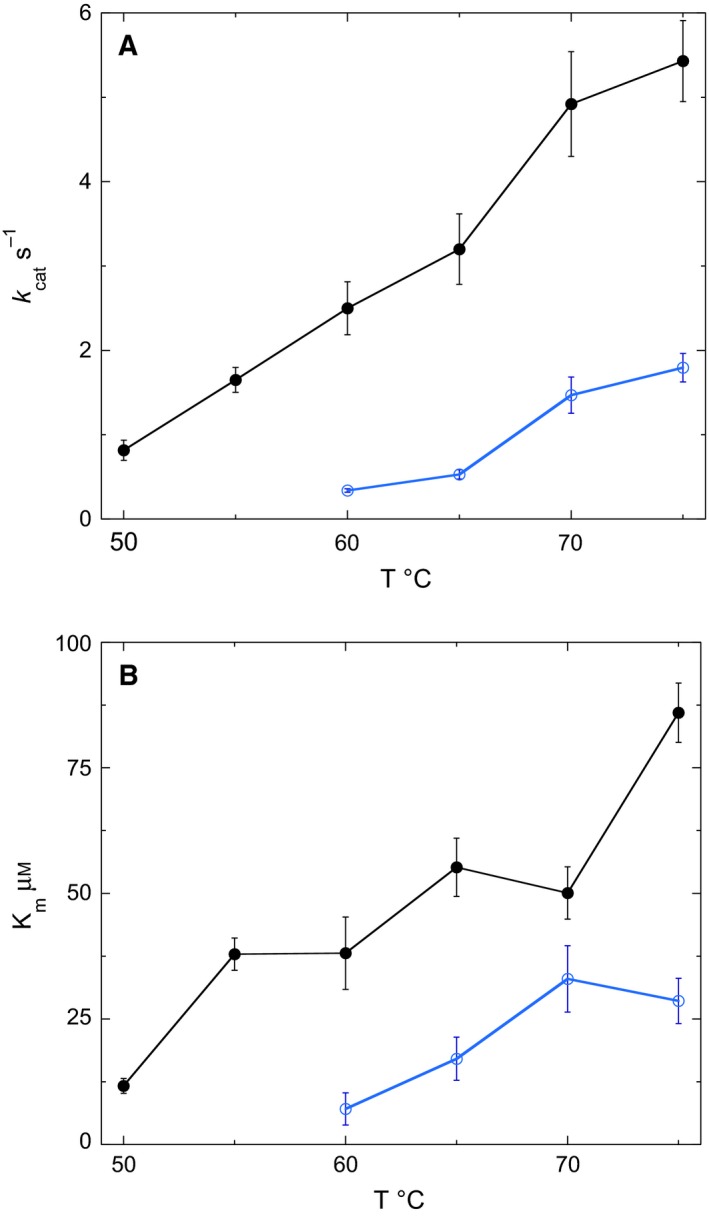
Temperature dependence of *k_cat_* (A) and *K_m_* (B) for wild‐type (blue) and quadruple mutant (black) *ph*CoADR kinetics with CoA disulfide as substrate. Three replicates were done in 50 mm potassium phosphate pH 7.5, with final concentrations (in the cuvette) of 110 μm NADH and 0.6–0.8 μm enzyme. Results show CoA disulfide is a substrate of the quadruple mutant as low as 50 °C, but the wild‐type enzyme has little activity below 75 °C.

To better understand how the mutations affected the activity the quadruple mutant, the temperature dependence CoA disulfide turnover was examined using NADH as the cosubstrate. The quadruple mutant actively reduced CoA disulfide with a *k*_cat_ much higher than the wild‐type enzyme over range of temperatures from 50 to 75 °C (Fig. 3A). The wild‐type enzyme shows consistently lower *K*_m_s for the CoA disulfide substrate than the quadruple mutant enzyme over the same range of temperatures (Fig. 3B).

The temperature dependence of wild‐type *ph*CoADR activity toward CoA disulfide suggests the active site of wild‐type becomes more flexible at higher temperatures to accommodate the substrate. The rate enhancement observed in the quadruple mutant and activity seen at lower temperatures may be due to the more open substrate channel and enhanced flexibility of residues 56–76 because of the Y65A, Y66A, and P67G substitutions. While the factors that contribute to the *K*_m_ value can be complex, it is not surprising that the quadruple mutant does not decrease the *K*_m_ for the CoA disulfide substrate. Widening of the substrate channel may allow for both easier, hence faster, substrate access to the interior of the enzyme, increasing the rate of E + S→ES. Likewise, the same mutations can promote a faster rate of substrate dissociation from the active site (ES→E + S). Hence, these mutations would not be expected to improve the binding specificity.

### Anaerobic NADH titrations of the quadruple mutant

**Figure 4:**
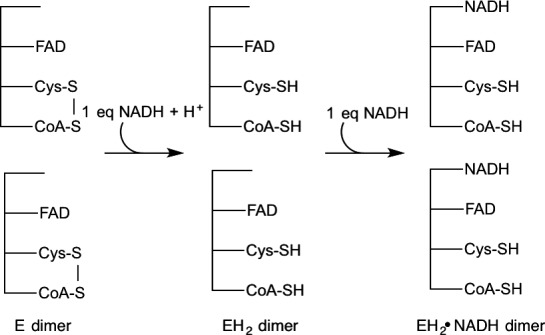
Scheme for anaerobic titration of wild‐type *ph*CoADR with NADH.

Previous anaerobic titrations of the wild‐type enzyme with NAD(P)H indicated the two subunits act in an identical manner whereby upon addition of 1 equivalent of NAD(P)H, the heterodisulfide composed of C48 and the bound CoA is reduced to the mixed disulfide form (Fig. 4) 8. Addition of excess NAD(P)H results in the formation of what appears to be the ‘EH_2_·NAD(P)H’ form of the enzyme, as indicated by the appearance of absorbance at > 500 nm. Spectral evidence clearly showed that the majority of the enzyme‐bound FAD remains oxidized, even in the presence of excess NAD(P)H. This spectral evidence is confirmed by the chemical detection of two free thiols in the EH_2_/EH_2_·NAD(P)H form of the enzyme, which reoxidize on exposure to O_2_.

**Figure 5:**
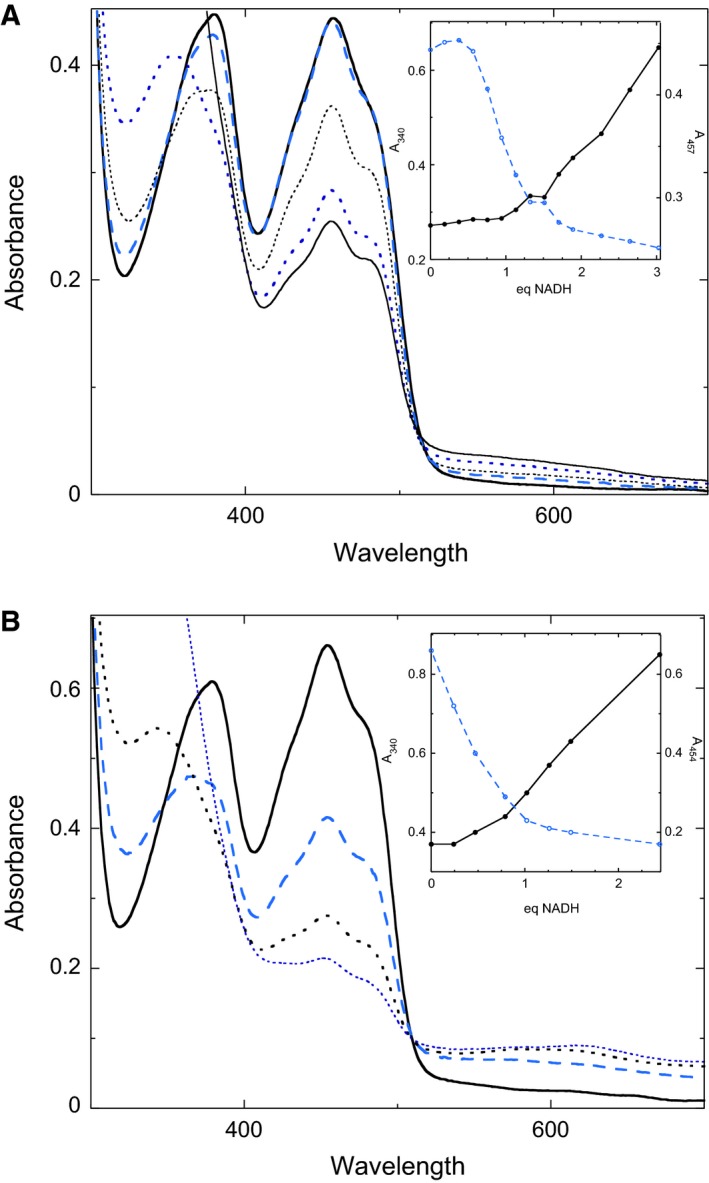
Anaerobic titration of *ph*CoADR with NADH. Titrations were performed in potassium phosphate, 50 mm, pH 7.5, at room temperature. (A) Quad mutant. Addition of 0 (—), 0.57 (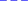), 0.95 (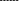), 1.51 (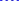), and 3.03 (—) eq NADH. Inset: Absorbance changes at 340 (——) and 454 (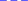) nm as a function of equivalents of NADH added. (B) Quad‐C48S Mutant. Addition of 0 (—), 0.47 (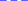), 1.02 (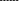), 2.44 (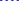) eq NADH. Inset: Absorbance changes at 340 (——) and 454 (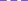) nm as a function of equivalents of NADH added.

**Figure 6:**
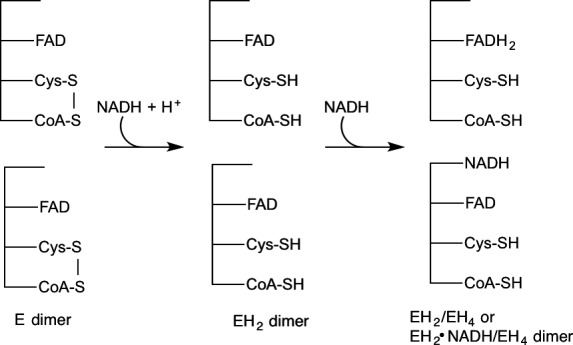
Scheme for anaerobic reduction of quad mutant phCoADR with NADH.

To test whether the four CoA‐binding pocket mutations altered the redox behavior of the enzyme, anaerobic titrations were performed with NADH. Titrating the first 0.5 equivalents of NADH per monomer to the quadruple mutant yielded little or no change in the UV–visible spectrum (Fig. 5A). Based on previous work on PNDOR enzymes, and titrations with the quadruple *ph*CoADR mutant containing an additional substitution that removes the active‐site cysteine (C48S) (*vida infra*), it is likely that the Cys‐S‐S‐CoA mixed disulfide was reduced in half of the active sites (Fig. 6), with the lack of an increase in absorbance at 340 nm demonstrating that the NADH was oxidized during this phase of the titration. This reduction of the active‐site disulfide does not show up spectrophotometrically in the absence of charge transfer absorbance at 550 nm 10, which was not apparent in the quadruple mutant.

Adding an additional 0.5 equivalents of NADH to each quadruple mutant monomer (to achieve a total of 1 equivalent of NADH titrated per active site) resulted in a decrease in the absorbance at 450 nm, which corresponds to nearly a 25% reduction of the enzyme‐bound FAD. This behavior stands in contrast to the wild‐type enzyme, where adding 1 equivalent of NADH per monomer reduces only the Cys‐S‐S‐CoA mixed disulfide and does not significantly affect the absorbance of FAD. This difference suggests the presence of a subunit asymmetry or half‐sites reactivity in the quadruple mutant not observed in wild‐type *ph*CoADR.

Upon addition of another 0.5 NADH equivalents, to achieve 1.5 total reducing equivalents of per monomer, 0.25 equivalents appear to reduce the active‐site FAD while the other 0.25 equivalents reduce the remaining active‐site disulfide bond. At up to three equivalents of NADH added per monomer FAD remains ~ 50% reduced, indicating reduction of the FAD on only one subunit in each dimer. During this second phase of the titration, a small increase in absorbance at > 500 nm is observed, which is most likely due to formation of an enzyme–NADH complex, an intermediate commonly observed in the PNDOR class of enzymes.

**Figure 7:**
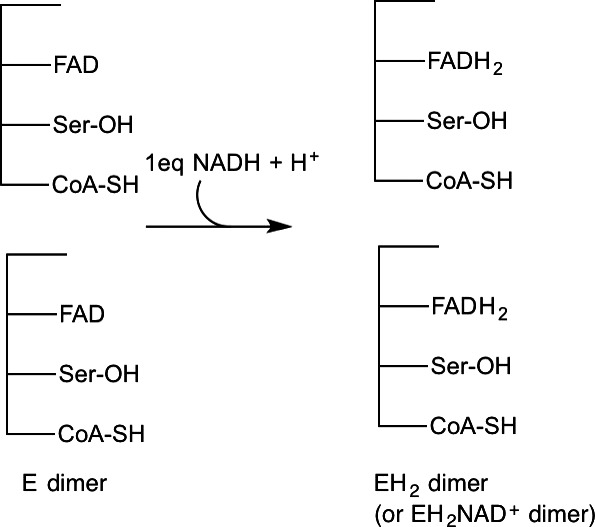
Anaerobic reduction of quad mutant phCoADR with NADH.

To verify that reduction of the active‐site cysteine was being observed during addition of the first 0.5 equivalent of NADH, active‐site Cys 48 was mutated to serine in the quadruple mutant, forming ‘quad‐C48S’. Anaerobic titration of the quad‐C48S mutant showed immediate reduction of FAD (Fig. 5B) and ultimately reduction of both FADs per dimer when just over 2 equivalents of NADH are added per dimer, as described in Fig. 7. This behavior stands in contrast to the quadruple mutant in which one FAD remained oxidized per dimer. It should be noted that in the fully reduced UV–vis spectrum of the quad‐C48S mutant, there is evidence of a weak charge transfer complex between FADH_2_ and NAD^+^ at λ_max_ = 600 nm. Because the absorbance at 600 nm remains with full reduction to FADH_2_, it does not appear that this broad long wavelength absorbance is due to the stabilization of a neutral FADH^·^ semiquinone intermediate, which is expected to have much stronger absorption features at both 570 and 630 nm.

## Discussion

### Substrate preference of PNDORs

From the crystal structure of *ph*CoADR, it is apparent that steric interference may inhibit accommodation of CoA disulfide in the active site. By increasing the volume of the CoA‐binding channel and flexibility of this site by making four mutations in *ph*CoADR (Y65A, Y66A, P67G, and H367G), we were able to increase the utilization of CoA‐S‐S‐CoA as a substrate across a range of temperatures. The mutations in the quadruple enzyme show that *ph*CoADR can be adjusted quite easily to accommodate the CoA disulfide substrate.

The lack of similar changes that reduce the size of the amino acid side chains in the wild‐type enzyme suggests there is little or no evolutionary pressure to develop an efficient CoA disulfide reductase in *Pyrococcus*. Because glutathione concentrations are low in most Archaea, an alternative small molecule should exist for maintaining cellular redox balance and combating oxidative stress. CoA disulfide is such a molecule in some organisms 15, 16, 17, but the poor CoA disulfide reductase activity of this enzyme suggests that the disulfide is not used for that purpose in *P. horikoshii* or that the CoA is used in a manner that does not require efficient reduction of the disulfide form, such as for a CoA‐per/polysulfide recycling system 4. Also present in *Pyrococcus furiosus* are a variety of antioxidant systems that operate independently of thiol/disulfides. These systems likely obviate the need for a glutathione or CoA‐dependent defense system 18.

### Impact of the quad mutations on the appearance of subunit asymmetry

Several members of the PNDOR family of enzymes show behavior indicative of intersubunit communication. Broadly, intersubunit communication is expressed in three general ways: the more frequently observed/well‐characterized forms of positive and negative cooperativity and the much less well‐understood form of half‐sites reactivity 19. In positive cooperativity, reaction velocities exhibit a sigmoidal increase with substrate concentration due to increasing affinity for substrate on subunits following the initial substrate binding to the first subunit. With negative cooperativity, binding at one site decreases substrate affinity on the other subunit(s) and also yields sigmoidal plot of substrate vs. velocity. With half‐sites reactivity on a dimeric enzyme, a reaction on one subunit prevents a reaction from occurring at the other subunit until a catalytic step or cycle is completed on the first subunit. Half‐sites reactivity can be difficult or impossible to detect via standard steady‐state kinetics, as velocity vs. substrate plots typically show ‘normal’ hyperbolic saturation kinetics even though the actual velocity of the enzyme is limited due to an asymmetry in the reactivity of the individual monomers. Rather than operating independently, each subunit fires in an alternating fashion, effectively reducing the *k*_cat_ of the enzyme to one‐half of the theoretical value expected in the absence of such asymmetry.

The PNDOR family of enzymes contains several members that exhibit half‐sites reactivity or similar asymmetry, including mercuric reductase 19, the NADH oxidase in *Enterococcus faecalis* 20 *,* and the CoADR in *S. aureus* 10. The subunits of the FAD‐ and NAD(P)H‐dependent mercuric reductase homodimer alternate between binding and catalysis 19. When one monomer is actively reducing mercury via NADPH, the other monomer is simply binding mercury and cannot participate in catalysis. Release of NAD(P)^+^ is required before activity commences on the alternate subunit.

Subunit asymmetry was also observed in the C42S mutant of NADH oxidase from *E. faecalis*. This homodimer reduces O_2_ to H_2_O, with the subunits alternating in activity 20. One monomer does not reduce O_2_ until the other completes the full reduction of O_2_ to 2H_2_O. In this case, the re‐oxidation of one monomer, rather than release of the oxidized dinucleotide, initiates the conformational change that allows the second subunit to commence activity. Finally, *S. aureus* CoADR exhibits asymmetry during reductive titrations. Addition of excess NADPH reduces FAD in one only subunit 10. The second subunit remains oxidized and bound to NADPH suggesting the reduction potential of FAD in this subunit shifted below that of NADH by the reduction of FAD on the first subunit. Half‐sites reactivity or subunit asymmetry is also seen on an array of enzymes unrelated to the PNDOR family, including glutamate dehydrogenase 21, aspartate transcarbamylase 22, and glyceraldehyde 3‐phosphate dehydrogenase 23.

It should be emphasized, however, that subunit asymmetry could, in many cases, be present on an enzyme but not expressed in an observable way. The PNDOR class of enzymes may be uniquely ‘good’ at revealing subunit asymmetry that may otherwise remain hidden because of the distinctive and informative spectra revealed during spectrophotometric reductive and oxidative titrations of the enzyme‐bound FAD.

For wild‐type *ph*CoADR, no obvious asymmetry was observed initially 8. During anaerobic titrations of the enzyme with NAD(P)H, both subunits acted in an identical manner and achieved the ‘EH_2_·NAD(P)H’ form of the enzyme when excess NADH is added (Fig. 4) 8. Anaerobic titration of the *ph*CoADR quadruple mutant with excess NADH, however, resulted in an asymmetric reduction of the protein (Fig. 6). One active site was reduced to the EH_4_ form (FADH_2_, 2RSH), while the second active site was found in the ‘EH_2_·NAD(P)H’ state. Based on these observations, the quadruple mutations likely have changed the reduction potentials of the FADs. To clarify, like the wild‐type enzyme, the active‐site disulfide bond in both subunits of the quadruple mutant is first reduced upon addition of NADH to make the 2EH_2_ form of the enzyme. Subsequent reduction of FAD in one EH_2_ active site by NADH results in EH_4_/EH_2_ but unlike the wild‐type enzyme, this shifts the potential of the FAD in the remaining EH_2_ site in the quadruple mutant low enough that it can no longer be reduced by NADH, instead leading to the EH_4_/EH_2_·NAD(P)H form of the enzyme.

It would be expected that the active‐site cysteine/CoA heterodisulfide on both subunits would be reduced by addition of the first eq of NADH (as in Fig. 4); the observed simultaneous reduction of FAD in one monomer and the remaining disulfide on the quadruple mutant (as shown in Fig. 6) was unexpected. In wild‐type enzymes in the PNDOR class in which the FAD is not reduced (but an FAD‐NADH complex is formed), the electron density in the C48 thiol is believed to prevent reduction of the adjacent FAD. In the quadruple mutant, the change in the active‐site structure results in change in the reduction potential such that FAD can be reduced after the initial reduction of the disulfide bond. The final product of the quad mutant titration is similar to the CoADR from *S. aureus* in which titration with excess NADPH yields reduction of only one monomer per dimer 10, although in *S. aureus* the FAD is reduced without the lag we saw in the quad mutant *ph*CoADR. *S. aureus* has a more open active site 4, 6, unlike the wild‐type *ph*CoADR but similar to the quadruple mutant.

Thus, the quadruple mutant introduces two significant changes: subunit asymmetry that was not apparent in the wild‐type phCoADR and a change in the reduction potentials of the redox centers such that one of the FADs in the dimer is reduced by NADH rather than remaining as an FAD‐NADH complex. One fairly obvious explanation for these changes may be a shift of the C48 thiol away from the FAD such that its potential is raised enough to allow for reduction by NADH. While the increase in flexibility of the substrate channel is consistent with this hypothesis, our structure does not allow us to make any concrete conclusions on this. Therefore, the cause of these changes is not known at this time, although these results do highlight the role of the enzyme in fine‐tuning the redox potential of the bound FAD to the potential(s) most conducive to catalysis.

### Function of subunit asymmetry/half‐sites reactivity

Future work with this NADH oxidase and the *ph*CoADR will explore the dimer interface in order to understand the communication between dimers that creates the asymmetry. The crystal structure of *ph*CoADR shows three residues from the other subunit stabilize the CoA substrate in the active site 4. In the *E. faecalis* NADH peroxidase, another member of the PNDOR family, the crystal structure reveals residues from the opposite subunit are integral in catalysis. Thus, there is a strong basis for intersubunit communication that might facilitate alternating subunit activity. While future work may reveal the mechanism of half‐sites reactivity, its purpose in many enzymes is still unknown. In the case of mercuric reductase, Miller *et al*. 19 suggest the alternating reactivity of the monomers might allow tight binding and sequestration of mercury in one subunit, while the other subunit reduces and then releases elemental mercury. In the case of the CoADR enzymes, it may serve to prevent ‘over‐reduction’ of the enzyme to the EH_4_ state in the absence of an oxidizing substrate, as the reduced FAD of the EH_4_ state should be sensitive to oxidation by O_2_, with the resultant formation of damaging H_2_O_2_.

## Conclusions

Mutation of four bulky and restrictive amino acids from the lining of the channel leading to the catalytic active‐site heterodisulfide on the *ph*CoADR to smaller amino acids resulted in a widening of the channel and an increase in the flexibility in that area of the structure. While the enzyme maintained its overall structure, the changes allowed for easier access of the bulky CoA disulfide substrate to the active site than seen with wild‐type enzyme, as evidenced by the increase in *k*_cat_ for the quadruple mutant, as well as the observation of CoA disulfide reductase activity at lower temperatures, temperatures at which this activity is not observable for the wild‐type enzyme.

These mutations also resulted in subtle shift in the redox behavior of the bound FAD such that reduction by excess NADH resulted in the formation of the asymmetric EH_2_·NADH/EH_4_ (or EH_2_/EH_4_) dimer rather than the EH_2_·NADH dimer observed upon NADH reduction of the wild‐type enzyme. This work suggests that half‐sites reactivity may be a consistent feature of CoADRs that is not always apparent in reductive titrations, but may be ‘hidden’ and acting only on the kinetic parameters of the enzyme in a way that is difficult to deconvolute.

## Author contributions

KS designed the mutagenesis experiments, overexpressed the enzyme, performed titrations of the enzyme with substrate and cofactor and performed kinetic experiments. KS also produced the first two drafts of the manuscript. JL performed titrations and kinetic experiments. DT performed kinetic experiments. BC performed the initial crystallization experiments. MHS oversaw the overexpression of the protein and designed experiments resulting in the crystalization of the protein and determination of its structure. EJC designed the overall experimental framework, designed the titration and kinetic experiments, and rewrote the manuscript.
